# ASTER-B regulates mitochondrial carotenoid transport and homeostasis

**DOI:** 10.1016/j.jlr.2023.100369

**Published:** 2023-04-06

**Authors:** Sepalika Bandara, Jean Moon, Srinivasagan Ramkumar, Johannes von Lintig

**Affiliations:** Department of Pharmacology, School of Medicine, Case Western Reserve University, Cleveland, OH, USA

**Keywords:** scavenger receptor class B type 1, gram-domain containing protein, β-carotene oxygenase-1, β-carotene oxygenase-2, lipids, membrane transport, carotenoids, vitamin A, mitochondria

## Abstract

The scavenger receptor class B type 1 (SR-B1) facilitates uptake of cholesterol and carotenoids into the plasma membrane (PM) of mammalian cells. Downstream of SR-B1, ASTER-B protein mediates the nonvesicular transport of cholesterol to mitochondria for steroidogenesis. Mitochondria also are the place for the processing of carotenoids into diapocarotenoids by β-carotene oxygenase-2. However, the role of these lipid transport proteins in carotenoid metabolism has not yet been established. Herein, we showed that the recombinant StART-like lipid-binding domain of ASTER-A and B preferentially binds oxygenated carotenoids such as zeaxanthin. We established a novel carotenoid uptake assay and demonstrated that ASTER-B expressing A549 cells transport zeaxanthin to mitochondria. In contrast, the pure hydrocarbon β-carotene is not transported to the organelles, consistent with its metabolic processing to vitamin A in the cytosol by β-carotene oxygenase-1. Depletion of the PM from cholesterol by methyl-β-cyclodextrin treatment enhanced zeaxanthin but not β-carotene transport to mitochondria. Loss-of-function assays by siRNA in A549 cells and the absence of zeaxanthin accumulation in mitochondria of ARPE19 cells confirmed the pivotal role of ASTER-B in this process. Together, our study in human cell lines established ASTER-B protein as key player in nonvesicular transport of zeaxanthin to mitochondria and elucidated the molecular basis of compartmentalization of the metabolism of nonprovitamin A and provitamin A carotenoids in mammalian cells.

Carotenoids, a class of yellow and red pigments, contain up to 15 conjugated double bonds (1). Pure hydrocarbon carotenoids are named carotenes, whereas their oxygenated metabolites are named xanthophylls. In humans, xanthophylls such as zeaxanthin and lutein play important physiological roles as blue light filters and antioxidants (2). Carotenes such as β-carotene are the precursor of the chromophore of visual pigments (3) and for the hormone-like compound retinoic acid that regulates gene expression via nuclear hormone receptors (4, 5).

Humans acquire the nutrients exclusively from the diet and distribute them in the blood in lipoproteins (6). The cellular uptake of carotenoid cargo from this transport vehicles is mediated by specific receptors, including the scavenger receptor class B type 1 (SR-B1 encoded by the *SCARB1* gene) (7). SR-B1 facilitates cholesterol and fat-soluble vitamins movement between high density lipoproteins and the plasma membrane (PM) (8, 9).

In cells, β-carotene oxygenase-1 and β-carotene oxygenase-2 (BCO1 and BCO2) convert carotenoids into apocarotenoid cleavage products (10). A regulated expression of BCO1 and BCO2 is critical for vitamin A production and carotenoid homeostasis (11, 12, 13). Furthermore, subcellular compartmentalization of the activities of the two different carotenoid cleavage enzymes prevents oxidative stress in mitochondria and reduces the risk of vitamin A deficiency (14).

Recently, we provided evidence that GRAM-domain containing 1 (GRAMD; also designated as ASTER) proteins bind carotenoids (15). The proteins were initially characterized in nonvesicular cholesterol transport and display a domain-like structure (16, 17). Their N-terminal GRAM domain interacts with phospholipids in the PM, the C-terminal transmembrane helix anchors the proteins in the endoplasmic reticulum (ER), and a VaST (ASTER) domain binds sterol and carotenoids (15, 17). We observed that xanthophyll accumulates in ASTER-B expressing tissues of mice, such as the adrenal glands and testis (15). Distinct expression patterns of BCO2 and ASTER-B in photoreceptors suggest that the proteins contribute to the characteristic distribution patterns of xanthophyll in the human retina (18). However, a direct demonstration that ASTER proteins facilitate transport of carotenoids between cellular membranes is lacking. Furthermore, it is not well defined whether the proteins display specificity for the binding and transport of carotenoids.

Herein, we employed *E. coli* strains that synthesize carotenes and xanthophyll to determine the binding of different types of carotenoids to the StART-like domain of ASTER-A and B. We purified the respective carotenoprotein complexes and characterized them spectroscopically. We established a cell-based test system to study the role of ASTER-B protein in nonvesicular carotenoid transport. We demonstrate that ASTER-B is critical for xanthophyll transport to mitochondria. In contrast, carotenes such as β-carotene did not accumulate in the organelles. Thus, we establish an ASTER protein–dependent sorting mechanism by which provitamin A (β-carotene) and nonprovitamin A (zeaxanthin and lutein) carotenoids are channeled into different metabolic pathways in cells.

## Material and methods

### Cell lines and culture conditions

Human A549 Cells and Human RPE (ARPE19) cells were obtained from American Type Culture Collection (ATCC). A549 cells were cultured in DMEM media constituted with 10% fetal bovine serum (Gibco) and 1% (v/v) antibiotic antimycotic (Gibco) at 37°C, 5% CO2. Human ARPE-19 cells were cultured in DMEM/F-12 (1:1) medium (Gibco) supplemented with 2.5 mM L-glutamine, 15 mM HEPES buffer, 10% fetal bovine serum (Gibco), and 1% (v/v) antibiotic antimycotic (Gibco) at 37°C, 5% CO2. Human ARPE19 cells were used from the passage 4–10 in all our experiments.

### Western blotting

Protein was extracted from cells using M-PER mammalian protein extraction reagent (Thermo scientific) with protease inhibitor (Thermo Scientific, Marietta, OH). Twenty-five microgram of protein per lane were denatured in loading buffer and subjected to SDS-PAGE. Primary antibody SR-B1 at the dilution of 1:1,000 (Abcam) and ASTER-B at the dilution of 1:500 (Proteintech) were incubated overnight in 4% BSA (Sigma Aldrich). Secondary anti-rabbit IgG antibody (Abcam) was incubated for 1 h at room temperature at 1:10,000 dilution. For confirming subcellular fractionation, markers for cytosol β-actin at the dilution of 1:2,000 (Cell signaling), mitochondria COX IV at the dilution of 1:1,000 (Cell signaling), and PM SR-B1 at a dilution of 1:1,000, and ASTER-B at a dilution of 1:500 were used as primary antibodies and incubated overnight in 4% BSA (Sigma Aldrich). Secondary anti-rabbit IgG antibody (Abcam) was incubated for 1 h at room temperature at 1:10,000 dilution. Western blots were scanned with the Odyssey Imaging System (LI-COR Biosciences) for chemiluminescence detection.

### qRT-PCR analysis

Total RNA was isolated from A549 and ARPE19 cells using TRIZOL reagent (Invitrogen, Carlsbad, CA). RNA concentration and purity were determined with a Nano-drop spectrophotometer (Thermo Scientific, Marietta, OH). cDNA was generated using the High Capacity RNA to cDNA kit (Applied Biosystems, Thermo Fisher Scientific, Waltham, MA). Gene expression analysis was carried out by real-time quantitative PCR using an Applied Biosystems Real Time PCR instrument with Taq Man probes (Applied Biosystems; Thermo Fisher Scientific, Waltham, MA). Primers used for analysis were human *GAPDH* (Hs99999905), *BCO2* (Hs01568558), *BCO1* (Hs01015939), *GRAMD1A* (Hs00385151), *GRAMD1B* (Hs01112371), and *GRAMD1C* (Hs00214023). Amplification was carried out using TaqMan polymerase Fast Universal PCR Master Mix (2×) No Amp Erase, UNG (Applied Biosystems; Thermo Fisher Scientific, Waltham, MA) following the manufacturer’s protocol. 20 ng cDNA was used per 10 μl reaction. Gene expression levels were normalized to the expression of housekeeping gene *GAPDH* using the ΔΔCt method as previously described (19).

### Immunocytochemistry and confocal imaging

A549 cells were grown on Labtek chamber slides (Nunc Thermofisher) for 24 h at 37°C in a 5% CO_2_ incubator. Cells were washed with 1× phosphate-buffered saline (PBS) and fixed with 4% paraformaldehyde (Electron Microscopy Sciences) for 30 min at room temperature. Then, cells were washed with PBST (PBS with 0.1% Triton X-100). The fixed cells were blocked with 4% goat serum for 1 h at 37°C and incubated with primary ASTER-B antibody B (1:200) at 4°C overnight and COX IV (1:500) at room temperature for 2 h. After a wash step with PBST buffer, cells were incubated with secondary Alexa flour rabbit 488 and Alexa flour mouse 555 (both in 1:500 dilution) at room temperature for 2 h. DAPI Fluoromount G (Southern biotech) was used to stain nucleus. Images were taken from the Leica HyVolution SP8 confocal microscope using the multiline argon laser and 405 nm diode laser with a ×63 C-Apochromat NA, 1.4-oil objective.

The same method was used for costaining of the ER marker Calnexin. First, A549 cells were incubated with Aster B (1:200) antibody as described above and then incubated with calnexin (1:250) antibody (Proteintech) at room temperature for 2 h. After a wash step with PBST buffer, cells were incubated with secondary Alexa flour rabbit 488 and Alexa flour mouse 594 (both in 1:500 dilution) at room temperature for 2 h. DAPI fluoromount G (Southern Biotech) was used to stain nuclei. Images were taken with an Olympus FV1200 Laser Scanning confocal microscope (Olympus America, Waltham, MA) using 405 nm diode laser for blue channel, 473 nm diode laser for green channel, and 559 nm diode laser for the red channel with an UPLXAPO100XO oil OFN26.5, NA1.45 objective.

### Protein expression, purification, absorption spectra, and HPLC analysis

Plasmids expressing murine maltose binding protein (MBP)-Aster-A and MBP-Aster-B were previously described (15) and transformed into zeaxanthin, β-carotene, and lycopene producing XL-blue *E. coli* cells (10). Protein expression, purification, absorption spectra measurement, and HPLC analysis were carried out as in previous protocols (15).

### Carotenoid uptake assay

A549 and ARPE19 cells were seeded in 100 mm plate and incubated at 37°C in 5% CO_2_ until they reached ∼90% confluency. Then, media were changed to serum-free media with 10 mM methyl-β-cyclodextrin (MCD) (Alfa Aesar/Thermo fisher) for 2 h at 37°C in 5% CO_2_. Serum-free medium was then mixed with 2 mM MCD and 2 μM zeaxanthin, lutein, or β-carotene dissolved in acetone (<1% v/v final concentration). The cells were incubated for 24 h under this condition to achieve cellular carotenoid uptake and transport. Cells were then washed with PBS for three consecutive times before they were collected by scraping and centrifugation. The collected cells were either immediately subject to the analysis or stored at −80°C until analysis.

### Subcellular fractionation and HPLC analysis

A549 and ARPE19 cells were grown at 37°C and 5% CO_2_. Cells were washed with PBS three times before collecting by scraping followed by centrifugation. The collected cells were fractionated using the protocol described in Sadler *et al.* (2016) (20). Briefly, cells were resuspended in HES buffer (250 mM sucrose, 20 mM HEPES, 1 mM EDTA, pH 7.4) with protease inhibitor (Thermo scientific) and homogenized using a 25-gauge needle and centrifuged at 600 *g* (Eppendorf benchtop centrifuge) for 5 min at 4°C. This step was repeated 2–3 times to ensure a complete breakdown of cells. Then, the resulting lysate was centrifuged 10,000 *g* (Eppendorf benchtop centrifuge) at 4°C for 20 min. The supernatant was collected as the cytosol fraction. The pellet was resuspended in protease inhibitor containing HES buffer and layered on high sucrose HES buffer (1.12 M sucrose in HES buffer, pH 7.4). This was followed by a centrifugation step at 41,000 *g* (Beckman Coulter, Optima Max-xp ultracentrifuge, TLA 100.3 rotor) at 4°C for 1 h. The mitochondrial fraction was pelleted at the bottom of the tube, and the PM fraction was layered on top of the high sucrose HES buffer layer. BCA (Thermo scientific) assays were used to estimate the protein concentration of the different subcellular fractions. Carotenoids were extracted from the different fractions with published protocols and subjected to HPLC analysis on a silica column using hexanes:ethyl acetate (70:30 v/v) as the mobile phase for zeaxanthin and hexanes: ethyl acetate (90:10 v/v) as the mobile phase for β-carotene (10, 21).

### MTT assay

Cell viability for each chemical used in the carotenoid uptake assay was measured by a MTT assay (CyQUANT MTT cell viable assay Thermo Scientific). In brief, Both ARPE19 and A549 cells were seeded in 96-well plate with appropriate media for 24 h. Next, these cells were washed with PBS for three times and replaced with serum-free media along with testing compound. After treatment, the cells were washed with PBS for three times and were incubated with 10 μl MTT solution for 4 h. This was followed by 100 μl of SDS-HCL solution for 4 h at 37°C. The amount of formazan dye was measured by detecting the absorbance at a wavelength of 570 nm with a microplate reader (Bio-Rad iMark Microplate Reader, USA). The compounds tested were acetone, MCD, zeaxanthin, and β-carotene.

### GRAMD1B si-RNA knockout in A549 cell line

Cells were seeded in 100 mm plate with DMEM medium with 10% fetal bovine serum and incubated in the 37°C and 5% CO_2_ incubator until cells reached the ∼80% confluency. TransIT-X2 dynamic delivery system (Mirus) was used to transfect the *GRAMD1B* Dsi-RNA (IDT) rArGrGrUrCrArGrArArArArCrUrUrArCrUrGrCrUrArGrUAC, rGrUrArCrUrArGrCrArGrUrArArGrUrUrUrUrCrUrGrArCrCrUrUrG. Cells were collected for the analysis after 72 h or cells treated with carotenoid as mentioned above carotenoid uptake assay.

### Statistical analysis

Data shown are the mean ± SD. Analysis was performed using unpaired two tail *t* test and one-way ANOVA using Graph pad Prism 8.0 software, and results were considered significant at ∗*P* < 0.05, ∗∗*P* < 0.005, ∗∗∗*P* < 0.0001.

## Results

### Binding properties of the StART-domain of ASTER proteins

**Fig. 1:**
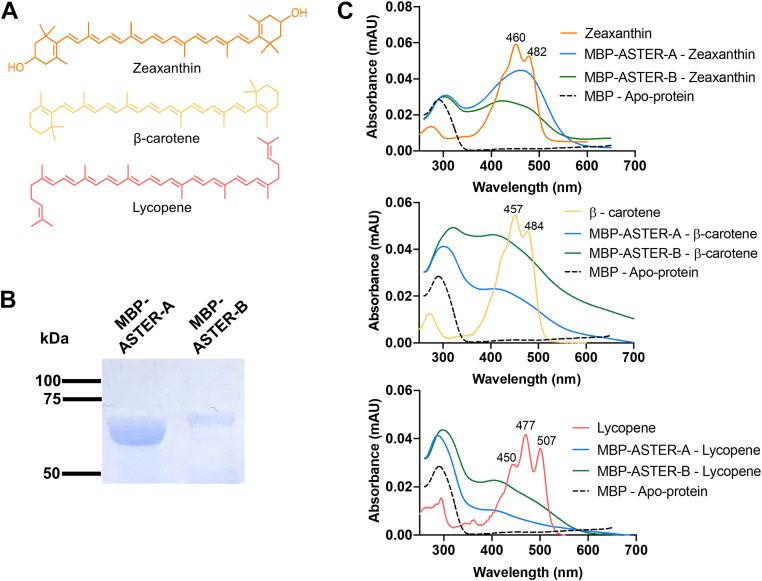
ASTER proteins prefer xanthophyll over carotene. A: Chemical structures of zeaxanthin, β-carotene, and lycopene. B: SDS-PAGE for the purified MBP-ASTER-A and MBP-ASTER-B proteins. C: UV-visible spectra of purified MBP-ASTER-A and MBP-ASTER-B proteins expressed in zeaxanthin producing *E. coli* (upper panel), β-carotene producing *E. coli* (middle panel), and lycopene producing (lower panel). The UV-visible spectra of the corresponding MBP-ASTER-A apoprotein and the protein unbound carotenoids are shown in the respective panels.

To test the binding specificity of the StART-like domains of ASTER-A and B, we expressed the proteins as recombinant MBP fusion proteins in bacteria that accumulate lycopene, β-carotene, and zeaxanthin, respectively (Fig. 1A). After expression, we purified the MBP-fusion proteins by affinity chromatography (Fig. 1B). We then recorded the spectral properties of the different carotenoprotein complexes and compared them to the purified recombinant apo-proteins and the free carotenoids (Fig. 1C). The purified MBP-ASTER-A and B proteins expressed in zeaxanthin-producing bacteria displayed two absorption maxima, one in the UV and a second maximum in the visible (Vis) range. The corresponding apoprotein showed only one maximum in the UV range (290 nm–320 nm) (Fig. 1C). The ratio of absorption between UV and Vis maxima was one to two for MBP-ASTER-A purified from zeaxanthin producing bacteria. This ratio was approximately equal for the MBP-ASTER-B carotenoprotein when purified from zeaxanthin-producing bacteria. The fine structure of the spectra of both carotenoprotein complexes was significantly different when compared to the free zeaxanthin, suggesting significant interactions between the chromophore of zeaxanthin and its binding proteins (Fig. 1C). MBP-ASTER-A carotenoprotein purified from β-carotene synthesizing bacteria displayed one to one absorption ratios between the UV and Vis maxima. However, the recorded Vis maximum revealed a significant bathochromic shift when compared to the Vis maximum of the zeaxanthin MBP-ASTER-A complex (Fig. 1C). The same was true for the MBP-ASTER-B though the ratio between the UV and Vis absorption was decreased. The MBP-ASTER-A and B preparation from lycopene synthesizing *E. coli* cells displayed a UV maximum but the absorption in the visible range was significantly lower when compared to the carotenoprotein complexes purified from zeaxanthin and β-carotene producing bacteria (Fig. 1C).

Previously, we extracted the bound carotenoids from MBP-ASTER-A and B and identified it as zeaxanthin (15). We now denatured the purified carotenoprotein complexes expressed in β-carotene and lycopene-producing bacteria and extracted the bound pigments. HPLC analyses revealed that lipid extracts of MBP-ASTER-A and B preparations from β-carotene synthesizing *E. coli* displayed a peak with the retention time and spectral properties of this carotene (supplemental Fig. S1A). In contrast, we detected no lycopene in lipid extracts of MBP-ASTER-A and B (supplemental Fig. S1A). Instead, carotenoids with spectral properties resembling intermediates of lycopene synthesis or lycopene degradation became detectable (supplemental Fig. S1B). HPLC analysis with carotenoprotein complexes of ASTER-A and B from zeaxanthin-producing *E. coli* detected β-cryptoxanthin and zeaxanthin in ASTER-A complexes and zeaxanthin in ASTER-B complexes (supplemental Fig. S1B, C)

Using the recently resolved structure of the StART-like domain of ASTER-A (17), we modeled zeaxanthin and β-carotene without any steric clashes into its lipid-binding cavity of the lipid-binding fold (supplemental Fig. S2). In contrast, the open chain lycopene did not fit the lipid binding cavity of ASTER-A. The in silico data supported the data from the expression analyses in bacteria and indicated that the StART-like domains of ASTER-A and B bind carotenoids with ionone rings such as β-carotene and zeaxanthin.

### A549 cells accumulate carotenoids in mitochondria

**Fig. 2:**
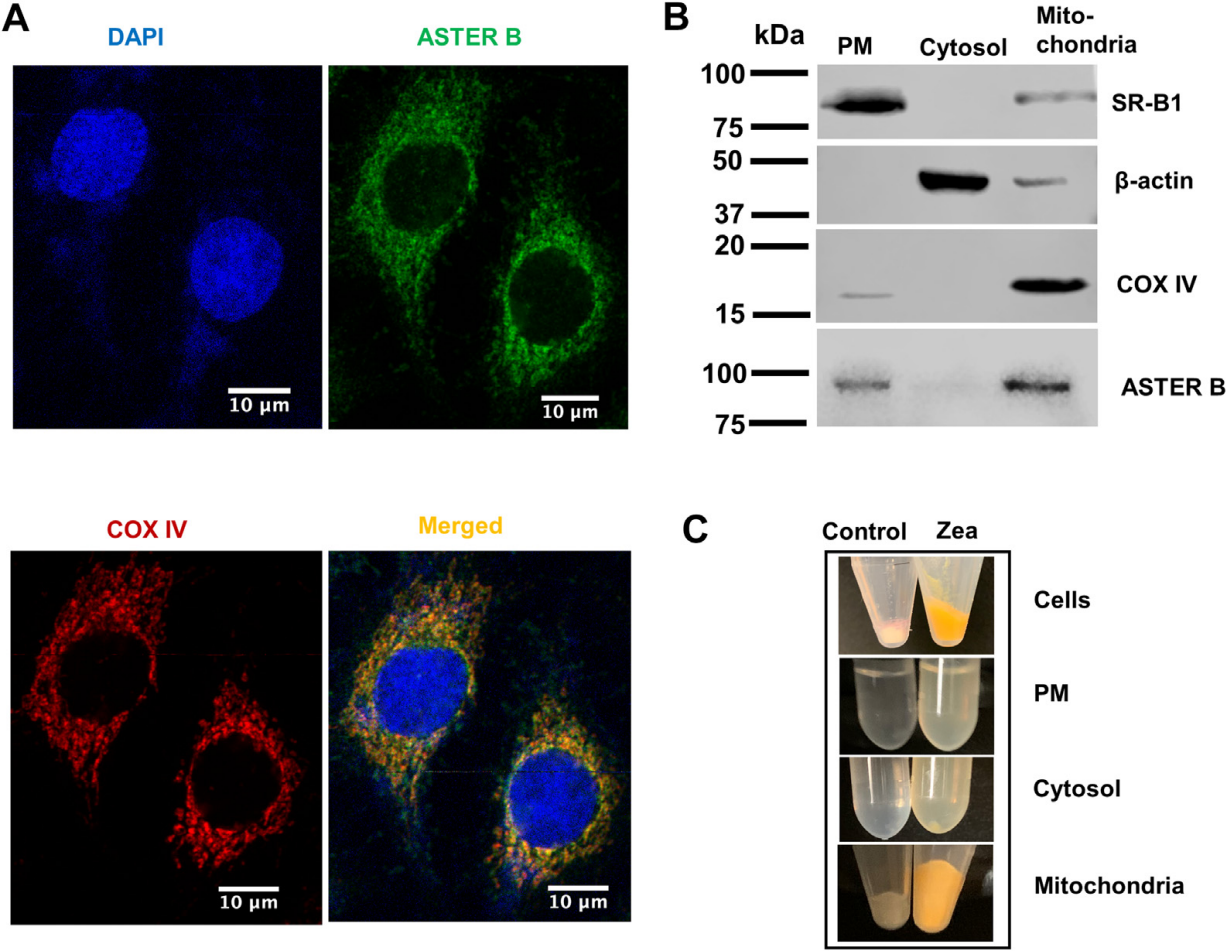
A549 cells express ASTER-B proteins and accumulate carotenoids in different cellular compartments. A: Immunocytochemistry of A549 cells stained with anti-ASTER-B (displayed in green) and anti-COXIV (displayed in red) antibody. Nuclei are stained with DAPI (displayed in blue). Merged images show orange color indicative for colocalization of ASTER-B and COXIV. The pictures are shown at 63× magnification. B: Western blot for marker proteins of SR-B1 (plasma membrane), β-actin (cytosol), COXIV (mitochondria), and ASTER-B. C: Colors of the different subcellular fraction of A549 cells incubated in the presence and absence of zeaxanthin (Zea) upon MCD treatment.

The human lung cancer cell line A549 expresses high levels of ASTER-B (15). We performed immunocytochemistry to analyze in which cellular compartment the protein was expressed in these cells. Confocal imaging showed a reticulated staining pattern for ASTER-B that spared the nucleus (Fig. 2A). We also stained the cells with an antiserum directed against cytochrome oxidase subunit IV (COXIV), a protein of the inner mitochondrial membrane. Merged images of ASTER-B and COXIV staining showed costaining in some areas of the cells as indicate by the orange color. This finding indicated that ASTER-B is associated with mitochondria (Fig. 2A) and confirmed the result of a previous study (22). Staining for Calnexin, an ER marker, and ASTER-B observed colocalization of the proteins (supplemental Fig. S3). Thus, our and pervious analyses (22) suggested that ASTER-B tethers ER and mitochondria to facilitate transport of carotenoids.

**Fig. 3:**
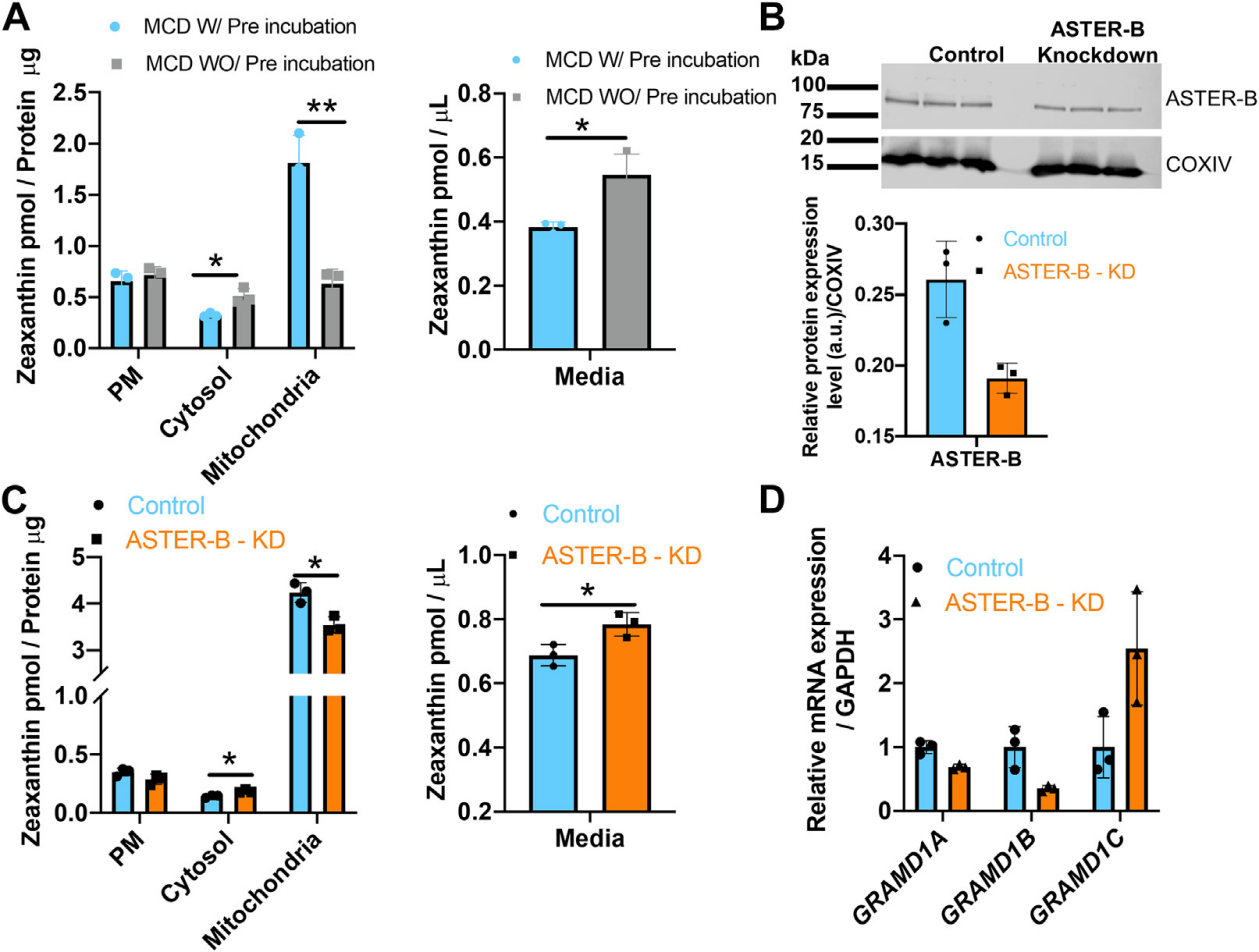
ASTER-B mediates zeaxanthin uptake in A549 cells. A: Zeaxanthin distribution in different cellular compartments of A459 cells and medium with and without pretreatment of MCD. B: Western blot for ASTER-B of protein extracts of A549 control cells and GRAMD1B knockdown (KD) A549 cells treated with GRAMD1B siRNA (100 nM) (Upper panel). Relative protein expression levels of ASTER-B in A549 cells quantified with the Image J software (Lower panel). C: Quantitative HPLC analysis of zeaxanthin concentrations in different cellular compartments and medium of A549 untreated (control) or siRNA-treated A549 cells (GRAMD1B-KD) cells. D: qRT-PCR analysis of *GRAMD1* gene expression of control and siRNA-treated cells. The RNA was isolated from A549 cells 72 h posttransfection with *GRAMD1B* siRNA. Data are displayed as the mean ± standard deviation of three independent experiments. Data were analyzed by unpaired two-tailed Student’s *t* test using Graph pad Prism 8.0 software. The data represents mean ± SD. ∗*P* < 0.05 and ∗∗*P* < 0.005.

To test this hypothesis, we established a cellular carotenoid uptake assay to assess the role of ASTER-B in carotenoid transport. Treatment of cells with MCD depletes the PM from cholesterol. Previously, fluorescent cholesterol metabolites have been utilized to demonstrate ASTER-B–dependent sterol transport from PM to the mitochondria (22). We now assessed the vehicles capacity to deliver carotenoid to cells and to stimulate the pigments transport to mitochondria (supplemental Fig. S4). Therefore, we incubated A549 cells in the presence of 10 mM MCD to deplete the PM from endogenous cholesterol as previously described (22). After the pretreatment, we incubated cells in medium containing 2 mM MCD and 5 μM zeaxanthin. As a control, we treated A549 cells without MCD-preincubation in the presence of medium containing 2 mM MCD and 5 μM zeaxanthin. Cell viability assays confirmed that treatment with MCD, solvents, and carotenoids did not affect A549 cell survival under the applied conditions (supplemental Fig. S5). We then harvested, lysed, and fractioned the cells into PM, cytoplasm, and mitochondria by an established protocol (20). The purity of the subcellular fractions was determined by Western blot for marker proteins, such as SR-B1 for the PM, β-actin for cytosol, and COXIV for the mitochondrial fraction (Fig. 2B). Western blot also revealed association of ASTER-B with the PM and mitochondrial fraction (Fig. 2B). Notably, all fractions showed a yellow color that is characteristic for carotenoids (Fig. 2C). We next extracted lipids from the individual fractions and performed quantitative HPLC analyses for zeaxanthin. In all cellular fractions, zeaxanthin became detectable (Fig. 3A). In cells pretreated with MCD, zeaxanthin was more than 3-fold enriched in mitochondria over the amounts in PM and cytosol. Interestingly, A549 cells without MCD pretreatment displayed lower zeaxanthin concentration in mitochondria. Additionally, the zeaxanthin concentration in cytosol was lower in these cells. The latter observation indicated that zeaxanthin is transported to mitochondria and that this transport is enhanced when the PM is depleted from cholesterol by MCD as previously described for sterols (17, 22, 23). The enhancement of zeaxanthin transport in MCD pretreated cells was also mirrored in a decreased concentration of zeaxanthin in the cell culture medium of A549 cells (Fig. 3A).

To demonstrate that mitochondrial zeaxanthin transport in A549 cells depends on ASTER-B, we performed loss-of-function experiments using siRNA treatment. Thus, we treated A549 cells with siRNA directed against *GRAMD1B* and siRNA control. We then subjected treated cells to MCD pretreatment followed by the MCD zeaxanthin uptake assay. Western blot analysis of differently treated cells showed that the siRNA treatment led to 50% decrease of ASTER-B protein (Fig. 3B). Quantitative HPLC analysis showed that mitochondria of *GRAMD1B* siRNA–treated cells contained less zeaxanthin than control cells. A similar decrease was observed in the cytosol of these cells. Accordingly, a higher concentration of zeaxanthin was found in the cell culture media (Fig. 3C).

The finding that siRNA-treated A549 cells still transported significant amounts of zeaxanthin can be explained by an incomplete knockdown of the translation of the target mRNA. Additionally, qRT-PCR analyses revealed that *GRAMD1C* mRNA expression was significantly increased upon siRNA treatment, suggesting that other ASTER protein variants compensated in part for the knockdown of *GRAMD1B* (Fig. 3D). Together, we established a novel MCD-based carotenoid uptake assay and showed that zeaxanthin accumulated in mitochondria of A549 cells.

### Comparison of carotenoid transport in A549 and ARPE19 cells

**Fig. 4:**
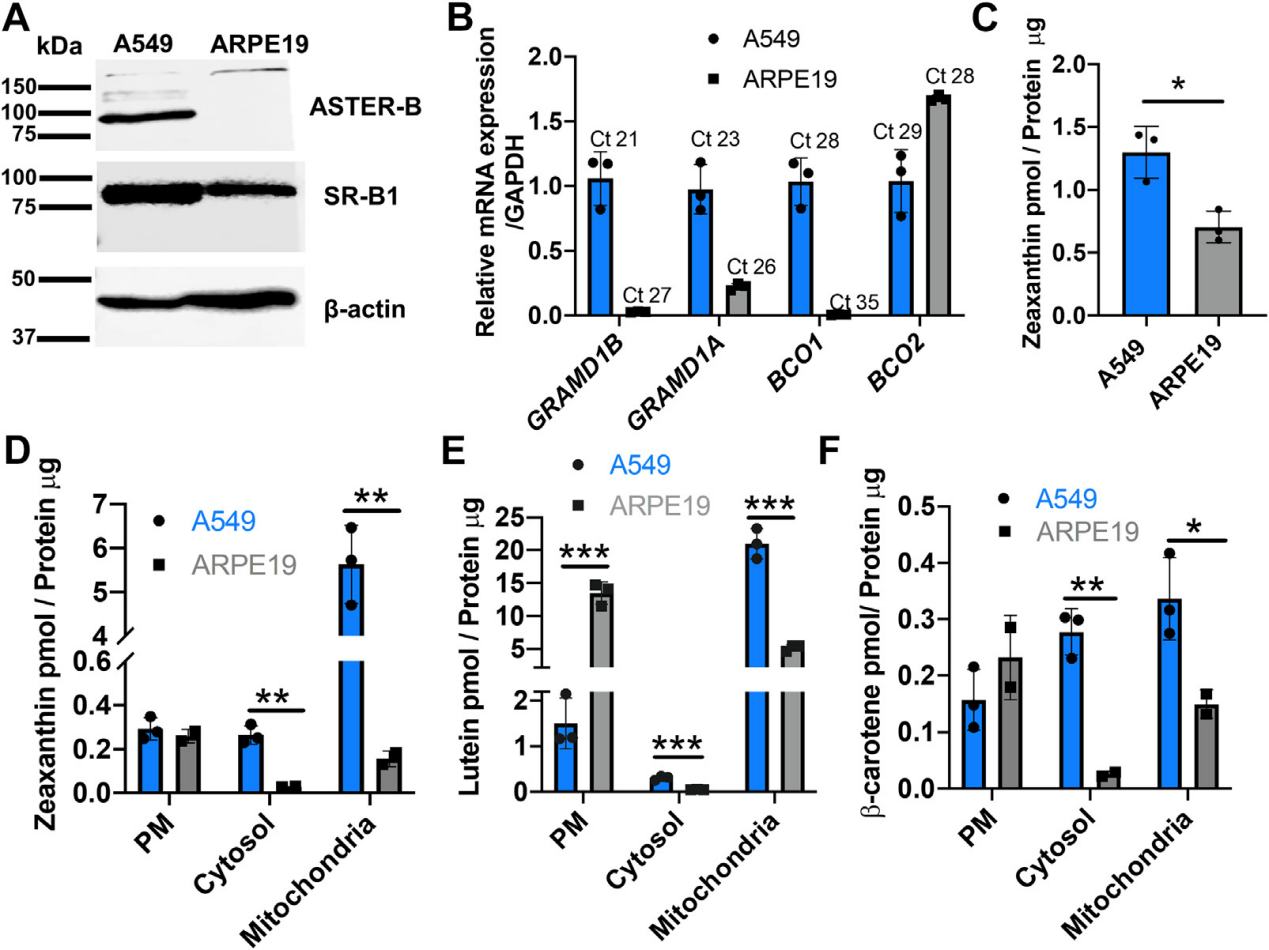
Comparison of carotenoid transport in A549 and ARPE19 cells. A: Western blots for SR-B1 and ASTER-B protein levels in A549 and ARPE19 cells. 25 μg of total protein was loaded per lane. B: qRT-PCR analysis for mRNA expression of *GRAMD1B* and *GRAMD1A* as well as *BCO1* and *BCO2* in A549 and ARPE19 cells. The graph displays the relative mRNA expression between the two cell lines. The Ct values for the individual genes are indicated in the figure. C: Zeaxanthin in ARPE19 and A549 cells upon the MCD-based uptake assay. D: Subcellular distribution of zeaxanthin in ARPE19 and A549 cells upon the MCD-based uptake assay. E: Subcellular distribution of lutein in ARPE19 and A549 cells upon the MCD-based uptake assay. F: Subcellular distribution of β-carotene in ARPE19 and A549 cells upon the MCD-based uptake assay. Data are displayed as the mean ± standard deviation of three independent experiments. Data were analyzed by unpaired two-tailed Student’s *t* test using Graph pad Prism 8.0 software The data represents mean ± SD. ∗*P* < 0.05 ,∗∗*P* < 0.005 and ∗∗∗*P* < 0.0001.

We next intended to compare carotenoid transport in A549 cells with a cell line that does not express ASTER-B protein. The human retina pigment epithelium cell line ARPE19 is routinely used for carotenoid uptake studies by other groups (7, 24). Western blot analysis revealed that A549 and ARPE19 cells both express the scavenger receptor SR-B1 (Fig. 4A). In contrast, ASTER-B expression was high in A549 cells but largely absent in ARPE19 cells. We performed qRT-PCR analyses to examine the expression of other members of the GRAMD gene family in ARPE19 cells. The analysis confirmed the absence of *GRAMD1B* gene expression, and we also observed a 5-fold lower expression of *GRAMD1A* mRNA when compared to A549 cells (Fig. 4B). Additionally, ARPE19 cells expressed no *BCO1* gene, whereas both A549 and ARPE19 cells showed mRNA expression levels of *BCO2* (Fig. 4B).

We employed the MCD-based uptake assay to study zeaxanthin transport in the two cell lines. After the incubation in zeaxanthin containing medium, we harvested cells by centrifugation, lysed them, and fractioned them into PM, cytoplasm, and mitochondria. The overall zeaxanthin uptake was two-fold higher in A549 when compared to ARPE19 cells (Fig. 4C). The PM fraction of the two cell lines displayed similar zeaxanthin content. In contrast, zeaxanthin was more than 40-fold enriched in the mitochondrial fraction of A549 when compared to ARPE19 cells (Fig. 4D). We next performed uptake assays with the carotenoid lutein (Fig. 4E). In ARPE19 cells, the highest lutein concentration was found in the PM fraction. In contrast, A549 cells amassed lutein 20-fold in the mitochondrial fraction when compared to the PM fraction (Fig. 4E). Collectively, the data suggested that ASTER-B in A549 cells facilitated the enrichment of zeaxanthin and lutein in mitochondria whereas the carotenoids were retained in the PM in ARPE19 cells.

Additionally, we analyzed β-carotene uptake in the two cell lines using the MCD-based protocol. We determined after fractionation into PM, cytosol, and mitochondria the concentration of β-carotene in A549 and ARPE19 cells (Fig. 4F).The mitochondrial accumulation of β-carotene in A549 cells was less pronounced (2-fold) than for zeaxanthin and lutein (>20-fold). In ARPE19 cells, β-carotene became detectable in PM and mitochondria, but there was again no enrichment in mitochondria. Thus, comparison between the two cell lines suggested that ASTER-B facilitates mitochondrial accumulation of zeaxanthin and lutein and that β-carotene transport is less influenced by ASTER-B.

## Discussion

Carotenoids and sterols are isoprenoids, meaning their carbon skeletons are built by the condensation of a distinct number of isoprene (C5) units. Their transport in the body is facilitated by the same lipoprotein classes, and their cellular uptake is mediated by the same lipoprotein receptors (6, 25). Moreover, both lipids are precursors for hormone-like metabolites which bind to ligand-activated transcription factors that belong to the superfamily of nuclear hormone receptors (26). Interestingly, critical steps in the metabolism of these lipids take place in mitochondria, particularly at the inner membranes of the organelles (27, 28). Recently, ASTER-B, a member of the GRAMD1 protein family, has been shown to facilitate the transport of cholesterol to mitochondria (17). ASTER-B displays a mitochondrial transfer sequence at its N terminus and deletion of the sequence or ablation of Arf1 GTPase which is required for mitochondrial translocation of ER proteins prevents mitochondrial cholesterol transport in cells (22). In our study, we confirmed the mitochondrial localization of ASTER-B by immunocytochemistry and confocal imaging in A549 cells. Moreover, we established novel tools and reagents to show that ASTER-B plays a role in the transport of carotenoids to mitochondria. The implications of our finding for carotenoid metabolism and function are discussed in the context of the current literature below.

Vertebrates acquire carotenoids exclusively from the diet and metabolically convert their chemical structures to generate a set of unique metabolites (6). A critical metabolite of carotenoids are apocarotenoids, including retinoids that derive from oxidative cleavage of distinct double bonds in the carbon backbone of the carotenoid molecules (29). Interestingly, major carotenoid modifying enzymes such as BCO2 exist at the inner mitochondrial membranes. However, it remained elusive for a long time how carotenoids are transported to mitochondria for metabolic processing through BCO2.

Mitochondria depend on the nonvesicular routes to receive lipids from the ER because they are disconnected from vesicular routes of lipid transportation (30). Recently, ASTER-B protein has been implicated in mitochondrial sterol transport. Accordingly, mice deficient of ASTER-B display impaired steroidogenesis in the adrenal glands (17). We previously showed that ASTER proteins bind carotenoids, but a role in carotenoid transport to mitochondria has not been established (15).

Mammalian genomes encode three *GRAMD1* genes (16). In our present study, we focused on ASTER-B, which displays a mitochondrial targeting sequence at the N terminus. The sequence is required for tethered ER and mitochondria contact sites to transfer sterols (22). We here speculated that carotenoid transport follows the same route. In order to study whether ASTER-B mediates the subcellular transport of carotenoids, we chose the A549 lung cancer cell line. As we previously showed A549 cells express high levels of ASTER-B protein (15). We established a novel MCD-based assay for carotenoid uptake in this cell line. MCD is known for interact with the PM and to deplete it from cholesterol (22). Therefore, we tested the correlation between MCD cholesterol depletion and carotenoid uptake, including its subcellular distribution into other cellular membrane systems. Preincubation of A549 cells with MCD significantly enhanced carotenoid accumulation into mitochondria when compared to A549 cells without pretreatment. The uptake and transport were reflected in a reduced carotenoid concentration in the media after the uptake assay in MCD-pretreated versus nontreated A549 cells. Furthermore, the subcellular distribution of the zeaxanthin to the mitochondria was affected by the preincubation of the cells with MCD. These findings suggested that cholesterol depletion of the PM increased the uptake of zeaxanthin as well as its accumulation in the mitochondria. By siRNA knockdown of *GRAMD1B*, we achieved a significant reduction of ASTER-B protein that was associated with a reduced accumulation of zeaxanthin in mitochondria. Though significant, the reduction of zeaxanthin was relatively mild. One contributor to the remaining uptake and transport of zeaxanthin under this condition might be the expression of other ASTER protein family members. GRAMD1C mRNA expression was low in A549 cells; however, the expression rose after knockdown of *GRAMD1B*. This finding suggested that ASTER-C might compensate in part for the reduction of ASTER-B protein upon siRNA treatment. The putative redundancy between different ASTER proteins is certainly an important issue of further research.

We used human ARPE19 cells to analyze whether ASTER-B is mandatory for carotenoid transport. Western blots revealed that this cell line expressed SR-B1 but not ASTER-B. ARPE19 did not show accumulation of zeaxanthin, lutein, and β-carotene in mitochondria though there was significant uptake of carotenoids into the PM of the cells. In contrast, zeaxanthin and lutein were amassed in the mitochondrial over the PM fractions in the MCD-uptake assays in A549 cells. We do not rule out that additional characteristics of ARPE19 and A549 cells affect carotenoid uptake and transport. However, the results from the siRNA experiments and the comparison between ARPE19 and A549 cells imply that ASTER-B facilitates the nonvesicular transport of carotenoids to mitochondria.

Interestingly, mitochondrial β-carotene accumulation was less pronounced than zeaxanthin and lutein accumulation in A549 cells. We cannot exclude that the MCD-based uptake assay has some limitations and favors xanthophyll over β-carotene uptake into cells. However, the finding that β-carotene is not amassed in mitochondria is consistent with our previous studies. β-Carotene is the substrate for both BCO1 and BCO2 enzymes in the test tube (10, 31). Nevertheless, β-carotene accumulates in mice deficient for BCO1 though the animals express significant amounts of mitochondrial BCO2 (32). In fact, studies in *Bco1* and *Bco2* knockout mice showed that zeaxanthin accumulated in mitochondria, whereas β-carotene did accumulate in lipid droplets of the cytosol (14). Accordingly, BCO1, the major β-carotene catabolizing enzyme, localizes to the cytosol, indicating that retinoids are exclusively produced in this cellular compartment (33). The expression of specific retinoid binding proteins in the cytosol may then facilitate their distribution within cells (34). In contrast, xanthophyll such as zeaxanthin and lutein are metabolized in mitochondria by BCO2, and ASTER-B may channel these hydroxylated carotenoids into this pathway. Thus, we propose that ASTER-B is a critical component for the compartmentalization of carotenoid metabolism. This compartmentalization preserves β-carotene for vitamin A production in the cytosol (14, 35) and channels xanthophyll to mitochondria.

## Data availability

The authors confirm that the data supporting the findings of this study are contained within the article and the supplementary information. The raw data are available upon request from the corresponding author.

## Supplemental data

This article contains supplemental data.

## Conflict of interest

The authors declare that they have no conflicts of interest with the contents of this article.
