# Supplementary material for: ASTER-B regulates mitochondrial carotenoid transport and homeostasis
Source: J Lipid Res. 2023 Apr 6;64(5):100369. doi: 10.1016/j.jlr.2023.100369 (PMC10193236; doi:10.1016/j.jlr.2023.100369)
Supplement: Supplemental Figures S1–S5 [file mmc1.pdf]

Supplemental information

ASTER-B regulates mitochondrial carotenoid transport and homeostasis

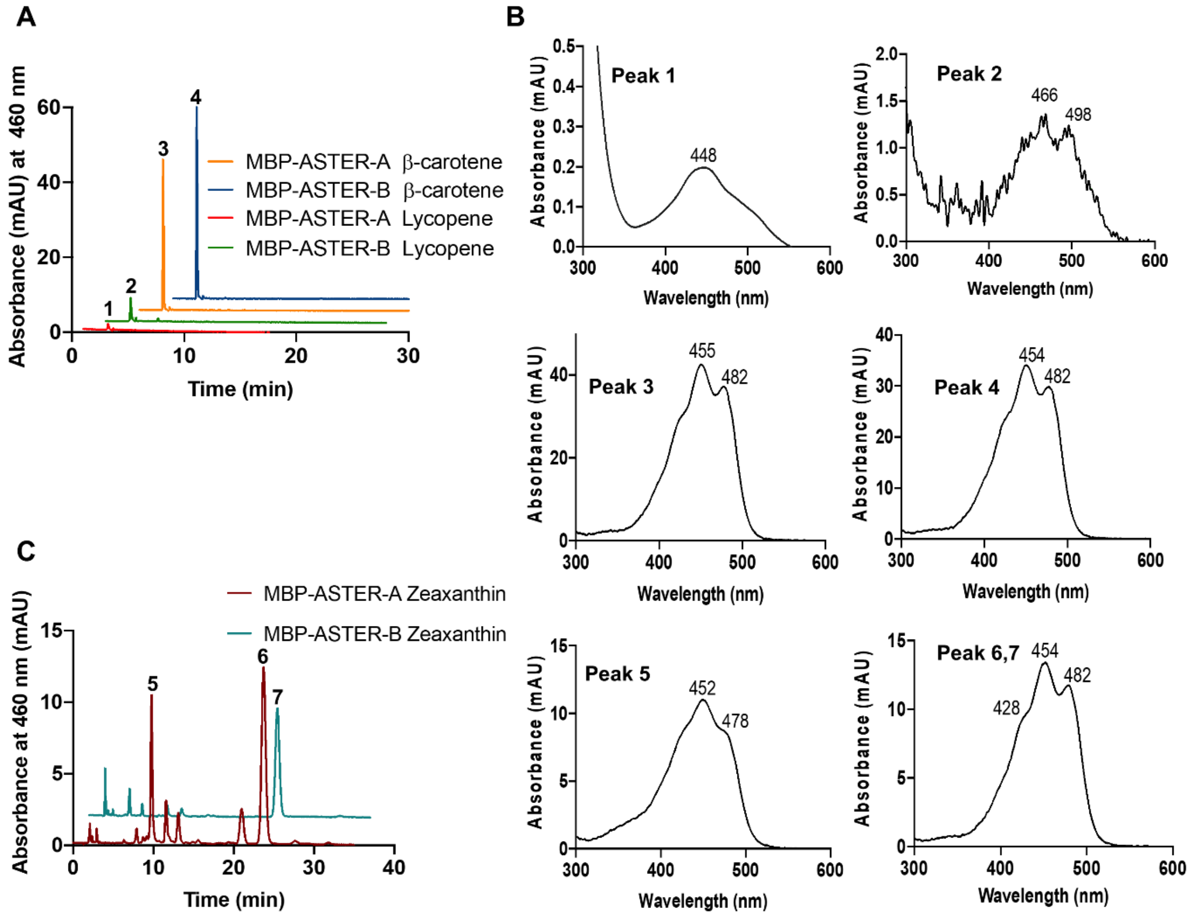

Figure S1 – **Analysis of carotenoids bound to MBP-ASTER-A and B. (A)** HPLC chromatogram at 460 nm of lipid extracts of purified MBP-Aster-A and B carotenoprotein complexes purified from β-carotene and lycopene producing *E. coli* cells. **(B)** UV-visible spectra of carotenoid peaks in the HPLC chromatogram. **(C)** HPLC chromatogram at 460 nm of lipid extracts of purified MBP-Aster-A and B carotenoprotein complexes purified from zeaxanthin producing *E. coli* cells. Peak 1, unknown carotenoids; peak 2, lycopene; peak 3 and 4, β-carotene; peak 5, β-cryptoxanthin; peak 7 and 8, zeaxanthin.

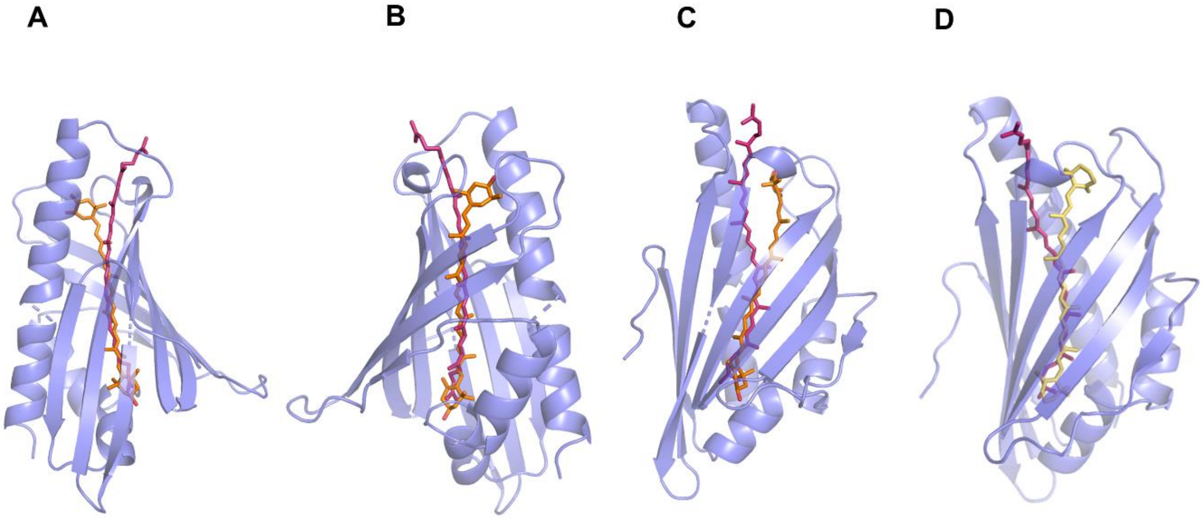

Figure S2 – (A-D) Three-side view (in 120° angle) of the structure of the StART-like (VaST) domain of murine ASTER-A (PDB ID – 6GQF) with a bound zeaxanthin (orange), lycopene (red), and β-carotene (yellow) modeled into the binding cavity.

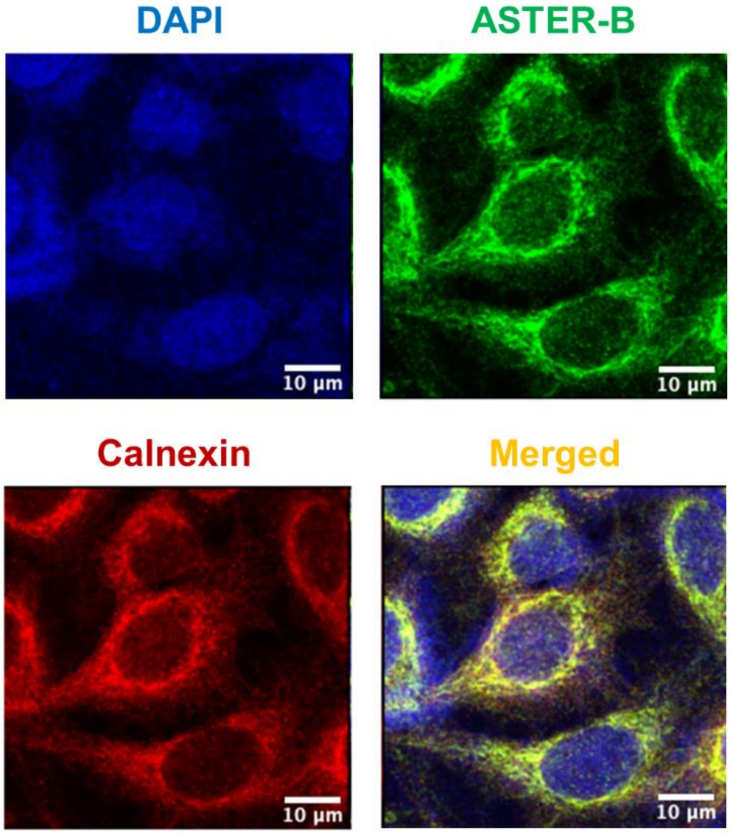

Figure S3. Immunohistochemistry of A549 cells stained with anti-ASTER-B (green) and anti-Calnexin (red) antibodies. Nuclei are stained with DAPI (displayed in blue). Merged images show yellowish color indicative for the co-localization of ASTER-B and Calnexin. The scale bar in the images indicate 10 μm.

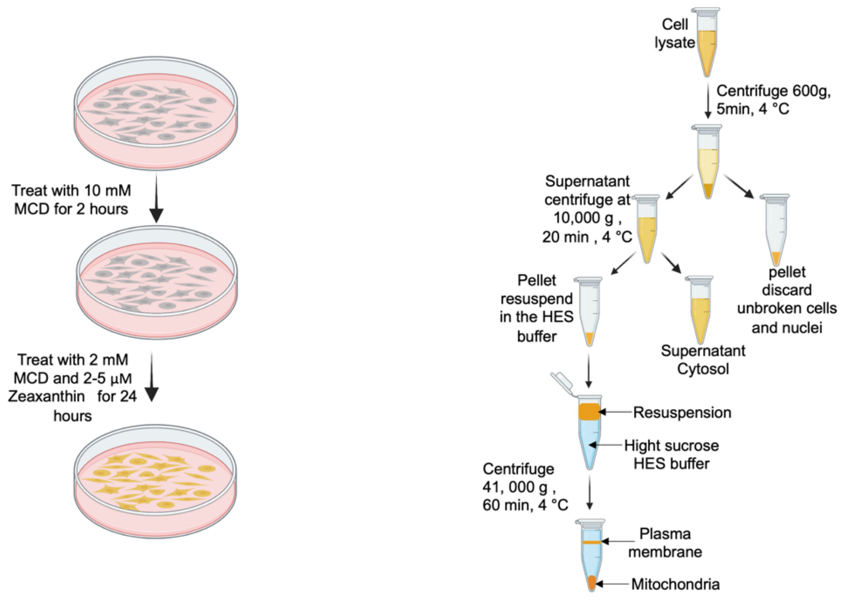

Figure S4 – Scheme of the MCD-based zeaxanthin uptake assay and subcellular fractionation.

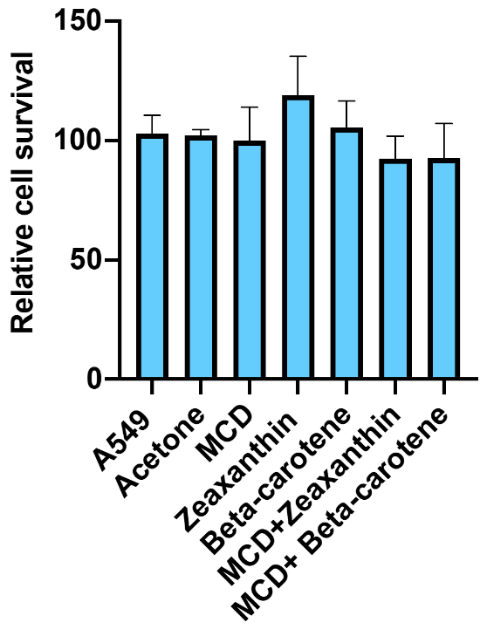

Figure S5 – Relative cell survival percentage after treatment of A549 with the individual components of the MCD-based carotenoid uptake assay. Survival is indicated in percent as compared to untreated cells. Data are display the mean +/- standard deviation of three independent experiments.
